# Application and prospects of somatic cell reprogramming technology for spinal cord injury treatment

**DOI:** 10.3389/fncel.2022.1005399

**Published:** 2022-11-17

**Authors:** Riyun Yang, Jingying Pan, Yankai Wang, Panhui Xia, Mingliang Tai, Zhihao Jiang, Gang Chen

**Affiliations:** ^1^Department of Histology and Embryology, Medical School of Nantong University, Nantong, China; ^2^Center for Basic Medical Research, Medical School of Nantong University, Nantong, China; ^3^Key Laboratory of Neuroregeneration of Jiangsu and the Ministry of Education, Co-innovation Center of Neuroregeneration, Nantong University, Nantong, China; ^4^Department of Anesthesiology, Affiliated Hospital of Nantong University, Nantong, China

**Keywords:** spinal cord injury repair, somatic cell reprogramming, fibroblasts, astrocytes, NG2 cells, neural progenitor cells, functional recovery

## Abstract

Spinal cord injury (SCI) is a serious neurological trauma that is challenging to treat. After SCI, many neurons in the injured area die due to necrosis or apoptosis, and astrocytes, oligodendrocytes, microglia and other non-neuronal cells become dysfunctional, hindering the repair of the injured spinal cord. Corrective surgery and biological, physical and pharmacological therapies are commonly used treatment modalities for SCI; however, no current therapeutic strategies can achieve complete recovery. Somatic cell reprogramming is a promising technology that has gradually become a feasible therapeutic approach for repairing the injured spinal cord. This revolutionary technology can reprogram fibroblasts, astrocytes, NG2 cells and neural progenitor cells into neurons or oligodendrocytes for spinal cord repair. In this review, we provide an overview of the transcription factors, genes, microRNAs (miRNAs), small molecules and combinations of these factors that can mediate somatic cell reprogramming to repair the injured spinal cord. Although many challenges and questions related to this technique remain, we believe that the beneficial effect of somatic cell reprogramming provides new ideas for achieving functional recovery after SCI and a direction for the development of treatments for SCI.

## Introduction

Spinal cord injury (SCI), a type of central nervous system (CNS) injury, can affect motor and sensory functions in the innervated area in mild cases and lead to paraplegia in worst-case scenarios. The incidence of traumatic spinal injury is 10.5 cases per 100,000 individuals worldwide (Kumar et al., 2018). SCI significantly affects the physical and mental health of patients, poses a substantial challenge to health care workers, and imposes a very large economic burden on the healthcare system (Algahtany et al., 2021). Therefore, efforts are required to improve patients’ status and quality of life and reduce the burden on families and society (Savic et al., 2018). SCI can be accompanied by complications such as respiratory and heart diseases, which are associated with a high mortality rate (Barbiellini Amidei et al., 2022). In the clinic, there are no effective methods for SCI treatment (de Araujo et al., 2022; Lee and Jeong, 2022).

**FIGURE 1:**
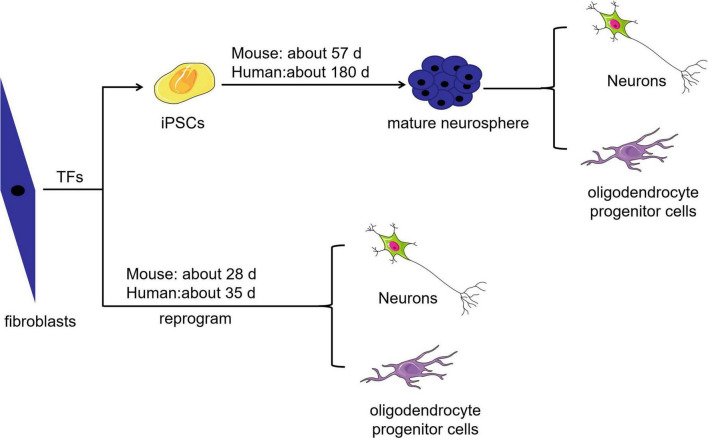
Comparison of the time required to obtain different target cells. The time required to obtain neurons and oligodendrocyte progenitor cells by somatic cell direct reprogramming is shorter than that required to obtain these target cells from iPSCs.

Cell-based regenerative therapy is a promising treatment strategy capable of modulating inflammatory responses and regenerating lost neural circuits (Papa et al., 2020). Researchers have integrated host circuits to aid the recovery of injured nerves in animals by transplanting autologous or homologous totipotent or multipotent cell-derived neurons (Lee and Jeong, 2022). Embryonic and neural stem cells are multipotent; the latter are more likely to differentiate into neural cells, but ethical concerns and limited supply restrict their clinical use. Although stromal stem cells can be easily used for autologous transplantation, their rate of transdifferentiation is low because these cells normally differentiate into mesodermal-lineage cells and must cross the germ cell layer to differentiate into neural lineage cells. In addition, there are some challenges related to mesenchymal stem cell use, such as a lack of standardized isolation protocols, the effect of cell heterogeneity on *in vitro* expansion, and the potential loss of multipotency during *in vitro* expansion (Hernandez et al., 2020). Induced pluripotent stem cells (iPSCs), the embryonic-like stem cells, can be induced from somatic cells treated with a cocktail of cytokines. iPSCs are convenient to use, have substantial developmental plasticity, can be used for autotransplantation, and can be induced to differentiate into neural stem cells. However, the clinical implementation of iPSCs remains complicated, and issues related to timing, influencing factors and the potential risk for teratoma formation need to be addressed (Csobonyeiova et al., 2019). As shown in Figure 1, mouse-derived somatic cells take 30 days to become iPSCs, 36 days to become embryoid bodies, 43 days to form immature neurospheres and 57 days to form mature neurospheres. It may take up to 180 days for human-derived somatic cells to become mature neurospheres (Matsui et al., 2014). Generally, the best time point for transplanting neural stem cells, the cells that derive from neurospheres, into SCI model animals is approximately 14 days after injury. However, the practicality of such a long-term experiment is unknown, and it seems doubtful that transplanted iPSC-derived neural stem cells can achieve the required rate of transdifferentiation (Kojima et al., 2019; Ahmadian-Moghadam et al., 2020; Giallongo et al., 2021).

Advances in iPSC- and transdifferentiation-related techniques have led to the development of somatic cell reprogramming approaches (Velychko et al., 2019). Somatic cell reprogramming skips the iPSC stage, reducing the time of transdifferentiation and the risk of teratoma development (Csobonyeiova et al., 2019). Some factors influence the efficiency of somatic reprogramming (Heinrich et al., 2015). For instance, studies have shown that reprogramming cells with the same embryological origin as neurons (i.e., the ectoderm) is an effective strategy for obtaining neurons (Zhou and Melton, 2008; Mattugini et al., 2019). Cell cycle status has also been shown to impact reprogramming. With advancements in biotechnology, somatic cell reprogramming has gradually become a feasible and promising strategy for neural repair, and it has been confirmed that neurons generated *via* this method can survive for a long time in the CNS. *In vivo*, reprogrammed neurons can form synaptic connections with primitive neurons and integrate into neural circuits, both locally and globally, ultimately facilitating the recovery of neural function (Wang F. et al., 2021).

Due to challenges related to CNS regeneration, there are still many difficulties to overcome before somatic cell reprogramming can be widely used in clinical practice. Currently, somatic cell reprogramming is the most appealing method for treating SCI. Several studies have revealed that the direct reprogramming of somatic cells into neurons and oligodendrocytes can effectively promote the recovery of motor function in SCI mice. In this review, we discuss the challenges of spinal cord repair and related advances in the direct reprogramming of somatic cells into neurons and oligodendrocytes using somatic cell reprogramming techniques.

## Pathophysiological characteristics of spinal cord injury

The interactions of many cell types, especially neurons, astrocytes, oligodendrocytes, and microglia, play an important role in spinal cord function. In SCI, these cellular interactions are disrupted, causing cell dysfunction and hindering the repair of the injured spinal cord (Anjum et al., 2020). SCI typically results from physical trauma, such as shearing, transection, and acute stretching/compression. SCI can cause a range of neurological symptoms leading to motor/sensory dysfunction, neuropathic pain, autonomic deficits, bowel/bladder dysfunction, and autonomic dysreflexia depending on the level and severity of the injury (Furlan et al., 2016).

SCI can be divided into four phases according to the time since the onset of injury, including the acute phase (< 48 h), subacute phase (48 h to 14 days), intermediate phase (14 days to 3 months), and chronic phase (> 3 months) (Tica et al., 2018). Acute SCI occurs when the spine is fractured and dislocated due to sudden trauma. The initial phase is the primary phase and is characterized by the presence of bone fragments and the tearing of spinal cord ligaments (Katoh et al., 2019). Regardless of the type of initial trauma, damage to the spinal cord blocks neural pathways, injures blood vessels, disrupts cell membranes, and leads to a range of pathophysiological consequences, including systemic hypotension, spinal shock, ischemia, vasospasm, neurotransmitter accumulation, and ionic imbalance (Ham and Leipzig, 2018; Chio et al., 2021). The severity of the initial damage and duration of spinal cord compression determines the consequence of SCI. After the primary injury occurs, a series of secondary events leading to chemical and mechanical injury in the spinal cord is initiated. In addition, loss of ion homeostasis following SCI causes an increase in the intracellular calcium ion concentration, and calcium overload eventually leads to cell death. Following SCI, the increased release of excitatory amino acids, such as glutamate, causes excitotoxicity, leading to neurological dysfunction (Vargas Luna et al., 2021). Secondary injury, the pathological process following primary injury, involves multiple phenomena. The initial clinical manifestations of secondary injury are hemorrhage and rapid death of necrotic cells, followed by regulated biochemical and molecular events, including vascular insufficiency, ischemia, neuronal excitotoxicity, the production of toxic free radicals, edema, dysregulation of ion homeostasis, lipid peroxidation, inflammatory reactions, immune reactions, astrocyte proliferation, demyelination, neuronal and glial apoptosis, glial scarring, and cyst formation (Anjum et al., 2020).

Cell death is a major mechanism of secondary injury (Karova et al., 2019). Initially, neurons become necrotic due to mechanical injury in the acute and subacute stages of SCI (Tang et al., 2021). Necrosis is caused by various factors, including the accumulation of toxic blood components, glutamate excitotoxicity, sudden loss of ion homeostasis, rapid loss of adenosine triphosphate (ATP), the production of proinflammatory cytokines by lymphocytes and neutrophils, and free radical formation (Alizadeh et al., 2019). Astrocytes are the most abundant cells in the CNS and are associated with SCI pathology. Primary injury induces astrocyte proliferation, which causes them to become reactive, leading to scarring (Yoshizaki et al., 2021). Subsequently, reactive astrocytes enter the injury epicenter, isolating inflammatory cells and preventing their spread (Gu et al., 2019), allowing fibroblasts and microglia to form fibrotic scars in the injury epicenter. Elongated reactive astrocytes and fibroblast-like pericytes interact with fibrotic scars to form glial scars (Dias et al., 2018). During spinal cord repair, dense glial scars act as barriers that inhibit nerve regeneration (Yokota et al., 2017). The persistence of systemic and local inflammatory responses from the acute to the chronic phase of SCI causes significant damage (Li et al., 2022). The death of cells that comprise neural circuits leads to the formation of cystic cavities known as spinal cavities (Berliner et al., 2020). These cystic cavities contain extracellular fluid, connective tissue, and infiltrating monocytes/macrophages (He et al., 2019), and an increase in cerebrospinal fluid pressure increases the volume of cystic cavities and hinders neural regeneration. These pathological and physiological responses are interrelated and interact with each other, making it challenging to alleviate SCI (Tran et al., 2018).

## Approaches for spinal cord injury treatment

There are three major challenges in the treatment of SCI. First, reactive glial cells respond rapidly, and because axons take time to regrow, scars formed by reactive glial cells can obstruct the growth of severed axons. Second, many injured neurons are lost due to neuronal death or apoptosis, and residual neural stem cells are insufficient to replenish these lost neurons. Third, reactive glia have both positive and negative effects, as they stimulate the release of neurotrophic factors, as well as cytokines that inhibit axonal regeneration (Chen et al., 2020). Various inhibitory factors interact at the injury site to form a chemical microenvironment unfavorable for axonal regeneration. As spinal cord repair is complex and affected by several factors, many challenges must be overcome to achieve neuronal replacement after nerve injury (Sofroniew, 2018; Carpenter et al., 2020; Li et al., 2020; Liu et al., 2020). Below, we discuss the classical methods for SCI treatment.

### Standard therapy

Researchers have used strategies involving identifying the type and site of specific injuries, removal of bone fragments and foreign bodies, and physical stabilization and decompression of the spine to treat SCI (Furlan et al., 2011). Additional approaches, including surgical decompression and administration of anti-inflammatory drugs, are needed to prevent, and treat complications (Oderich, 2021). Furthermore, physical rehabilitation therapy, such as exercise and gait training, is beneficial for repair of the injured spinal cord (Gomara-Toldra et al., 2014). The abovementioned therapies are used in clinics. Currently, the ability of non-pharmacologic approaches, including hypothermia treatment (Tracy et al., 2015) and cerebrospinal fluid drainage (Kwon et al., 2009), to reduce post-SCI inflammation is being investigated in clinical trials with promising early results (Ashammakhi et al., 2019b).

### Glial scar therapy

Some studies have demonstrated that moderate inhibition of glial scar formation can promote functional recovery after SCI (Bradbury and Burnside, 2019; Yang et al., 2020b). For example, researchers have used multiple genetic loss-of-function strategies to decrease the number of scar-forming astrocytes and promote neuronal regeneration in adult mice after SCI (Rodriguez et al., 2014; Wu et al., 2021).

### Stem cell therapy

Stem cell-based therapies, including embryonic stem cell, stromal stem cell, neural stem cell and iPSC therapies, are promising approaches for spinal cord repair (Cusimano et al., 2018; Ashammakhi et al., 2019b; Shang et al., 2022). For instance, the transplantation of iPSC-derived neural stem cells into damaged tissues after SCI was found to have neuroprotective effects and promote neural regeneration (Shang et al., 2022). In a rodent model of SCI, transplantation of human iPSC-derived neural stem cells was found to promote the expression of synapse-related genes and proteins in the area surrounding the injured site and prevent atrophy of the injured spinal cord, thus promoting the recovery of motor function (Kawai et al., 2021). However, this strategy has limitations, such as a low cell survival rate and the possibility of uncontrolled differentiation after stem cell transplantation (Duncan et al., 2020).

### Somatic cell reprogramming therapy

**FIGURE 2:**
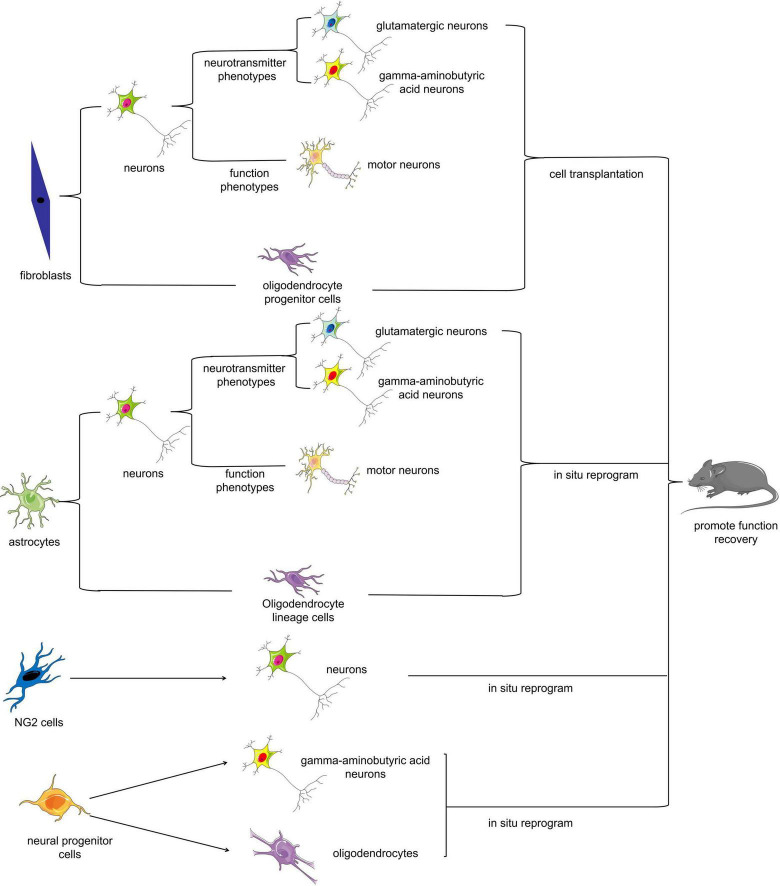
Cell fate reprogramming in the mammalian spinal cord. Multiple cell types can be reprogrammed either *in vitro* or *in vivo*. Fibroblasts, astrocytes, NG2 cells, and neural progenitor cells can be reprogrammed into neurons or oligodendrocytes, which can ultimately improve functional recovery after SCI.

Somatic cell reprogramming therapy mainly involves reprogramming somatic cells that can be easily isolated or proliferate extensively upon nerve injury (e.g., fibroblasts or reactive astrocytes) into target cells such as neurons by biotechnological means *in vitro* or *in vivo* (Figure 2). There are two potential approaches for utilizing somatic cell reprogramming for the treatment of SCI: endogenous repair and exogenous repair. Somatic cell reprogramming can generate cells for transplantation from endogenous or exogenous cell sources. Many recent studies have revealed that fibroblasts and astrocytes can transform into motor neurons *in vitro* (Son et al., 2011; Abernathy et al., 2017; Qin et al., 2018; Zhou et al., 2021). Some scholars have successfully transplanted these reprogrammed motor neurons into SCI model animals; others have reprogrammed somatic cells directly into neurons *in situ* using biotechnological means, all with excellent results (Zarei-Kheirabadi et al., 2019; Lee et al., 2020; Yang et al., 2020b).

### Biomaterial therapy

Conduits, scaffolds, fibers, sheets, hydrogels and particles can be used as biomaterial therapies for SCI (Ashammakhi et al., 2019b). The use of scaffolds (Guest et al., 2018), conduits (Ziemba and Gilbert, 2017), and fibers (Li et al., 2015) can guide axon regeneration. Implanting the Neuro-spinal Scaffold™ comprising poly(lactic-co-glycolic acid)-b-poly(L-lysine) into a thoracic SCI in rats and minipigs diminished inflammation and reduced the lesion cavitation volume in preclinical studies (Guest et al., 2018). The Neuro-spinal Scaffold™ is currently being evaluated in clinical trials (Theodore et al., 2016). Small-scale clinical trials showed promising results after Neuro-spinal Scaffold™ therapy for thoracic SCI (Theodore et al., 2016). The biomaterial polyethylene glycol (PEG) has been approved by the US Food and Drug Administration for many applications. PEG hydrogels have been found to decrease glial scar invasion and support and guide axon regeneration in SCI repair (Kong et al., 2017; Dumont et al., 2019). Intravenous injection of ferulic acid-glycol chitosan nanoparticles could promote functional recovery in SCI rates (Wu et al., 2014). Three-dimensional (3D) magnetic hyaluronic acid hydrogels with biochemical and biophysical properties similar to those of the spinal extracellular matrix have been developed to promote the growth of functional neurons and the expression of excitatory and inhibitory ion channels (Tay et al., 2018). Hyaluronic acid hydrogels containing extracellular matrix from mouse embryonic stem cell-derived astrocytes were shown to decrease the size of the glial scar after SCI and promote axonal growth through the injury site, demonstrating their therapeutic potential (Thompson et al., 2018). A low-elastic hydrogel with an aligned nanofiber structure was found to promote axonal regeneration and motor function recovery in SCI rats (Yao et al., 2018).

### Combinatorial therapy

Combinatorial treatments have been developed based on the complex pathophysiological changes after SCI. These approaches involve the delivery of a combination of biomaterials and cells or soluble molecules to the site of injury (Hou et al., 2022). The combination of biomaterial scaffolds and the transplantation of mesenchymal stem cells promotes the formation of connections between multiple cells during the pathological process after SCI (Papa et al., 2020). Hydrogels comprising hyaluronic acid mixed with methylcellulose can deliver cells to the injured spinal cord. Studies have shown that using such carriers for transplanting neural stem/progenitor cells effectively promotes behavioral recovery in SCI rats (Mothe et al., 2013). Hydrogels can be used as slow-release small-molecule drug reservoirs (Wang et al., 2022). For instance, serpin delivered by a chitosan-collagen hydrogel demonstrated a prolonged therapeutic effect and facilitated functional recovery in SCI rats (Kwiecien et al., 2020). Combinatorial therapies have also shown promise in promoting neurotrophic factor expression, optimizing the microenvironment after traumatic injury, improving cell survival after implantation, and promoting neuronal differentiation, neurite extension and linear axonal regeneration (Lin et al., 2016; Ashammakhi et al., 2019a).

Overall, significant preclinical advances related to spinal cord repair have been made, but these strategies should be further validated before widespread clinical application. Compared with other therapies, the advantage of somatic cell reprogramming therapy is that it cannot only provide cells for the transplant of exogenous somatic cell-derived neurons or oligodendrocytes for SCI repair but also replenish new neurons or oligodendrocytes at injury sites from neighboring endogenous glial cells (Li and Chen, 2016). Moreover, astrocytes can be targeted *in vivo* using viral vectors with specific promoters (Wang and Zhang, 2018). It is well known that glial scars play both inhibitory and protective roles in SCI repair (Yang et al., 2020a). Reducing the number of reactive astrocytes that proliferate and induce negative effects after SCI while effectively supplementing the number of neurons (including motor neurons with corresponding functions) at the site of injury is a promising method to address the challenges of nerve regeneration after SCI. This approach could appropriately eliminate glial scars and replenish lost neurons *in situ* while also improving the neural regeneration microenvironment. In conclusion, the potential of somatic cell reprogramming therapy in SCI provides new ideas for the development of cell supplementation and personalized regenerative medicine strategies.

## The sources and biological characteristics of somatic cells for reprogramming

Multiple somatic cell types can be reprogrammed *in vitro* or *in vivo* (Wang and Zhang, 2018). These somatic cells, including fibroblasts, astrocytes, NG2 cells, and neural progenitor cells, can all be reprogrammed into neurons or oligodendrocytes for spinal cord repair. Fibroblasts are widely distributed in connective tissues (Xu and Yao, 2021) and throughout the body and perform various functions, such as producing cytokines, growth factors and extracellular matrix components (Derk et al., 2021). Fibroblasts are easy to obtain, which is an important advantage for reprogramming strategies. Although fibroblast-derived neurons or oligodendrocytes are not used for *in situ* reprogramming, these cells can be used as a source of cells for transplantation for SCI repair (Heinrich et al., 2015). Astrocytes rapidly activate and proliferate after SCI, leading to the formation of a glial scar around the injured area. Reactive astrocytes display high expression levels of the neural stem cell markers Nestin and Vimentin (Sofroniew, 2015). After SCI, the reprogramming of reactive astrocytes *in situ* not only replenishes neurons or oligodendrocytes in the injured spinal cord but also modestly reduces the density of the glial scar (Ding et al., 2021; Yang et al., 2023). These characteristics of astrocytes make them appealing targets for reprogramming strategies for SCI repair (Chen and Li, 2022). NG2 cells, also known as oligodendrocyte precursor cells, are the fourth type of glial cells in the CNS, in addition to astrocytes, oligodendrocytes, and microglia. NG2 cells constitute a circulating population of glial cells in the adult CNS. These cells can rapidly proliferate and differentiate and are readily replaced after glial cell injury (Kirdajova and Anderova, 2020). NG2 cells are a good source of cells for reprogramming due to their proliferative capacity (Wang and Zhang, 2018). Neural progenitor cells are a heterogeneous population of adult cells with different differentiation capacities and multidifferentiation potentials. Although the number of resident neural progenitor cells is small after SCI, the multidifferentiation potential of these cells indicates their potential as a cell source for reprogramming.

In summary, multiple somatic cell types can be used for reprogramming for SCI repair. However, it is critical to consider the use of somatic cell reprogramming for the functional recovery of SCI. In the following sections, we discuss the application of somatic cell reprogramming strategies for repair of the injured spinal cord.

## *In vitro* fibroblast reprogramming and *in vivo* transplantation

Fibroblasts can be reprogrammed into other cell types, including adipocytes and chondrocytes (Dorrier et al., 2022). Thus, fibroblasts have an important advantage for reprogramming because they are easy to obtain and demonstrate multidifferentiation potential. Researchers have successfully transformed fibroblasts into cardiomyocytes (Bektik and Fu, 2021), hematopoietic progenitor cells (Silverio-Alves et al., 2019), neurons, motor neurons (Son et al., 2011; Qin et al., 2018; Hu et al., 2019), dopaminergic neurons (Li et al., 2019), glutamatergic neurons, GABAergic neurons (Vierbuchen et al., 2010) and oligodendrocyte progenitor cells (Lee et al., 2018). However, fibroblasts have been found to be involved in fibrotic scar formation following acute CNS injury (Derk et al., 2021), indicating their importance in injured spinal cord repair. In the following section, we elaborate on the effectiveness of fibroblast reprogramming strategies in spinal cord repair (Table 1).

**TABLE 1:** Recent studies on the potential therapeutic effect of fibroblast reprogramming on SCI.

Subject	Induction method	Cell type	Achievements	References
Rat	Small molecules (CHIR99021, forskolin, LDN193189, SB431542, SP600125, VPA, and Y-27632)	Neurons	Fibroblasts were converted to neurons *in vitro*; a neural network on a 3D porous filament scaffold, i.e., a neural scaffold was formed; transplantation of this neural scaffold into rats with spinal cord transection promoted motor recovery and axonal regeneration.	Hu et al., 2019
Human	Alternating administration of two combinations of small molecules to the medium (Combination I: CHIR99021, LDN193189, RG108, dorsomorphin, P7C3-A20, A83-01, and ISX9; combination II: forskolin, Y27632, DAPT, PD0325901, A83-01, PMA, and P7C3-A20)	Glutamatergic neuron-like cells	Fibroblasts were converted to glutamatergic neuron-like cells *in vitro* (Based on transcriptional profiles, electrophysiological function, and synaptic activity)	Yang et al., 2019
Mouse	Lentivirus-mediated transcription factors (Ascl1, Pou3f2, Myt1l)	Neurons (including glutamatergic neurons and GABAergic neurons)	Fibroblasts were converted to glutamatergic neurons and GABAergic neurons *in vitro*	Vierbuchen et al., 2010
Human	Lentivirus-mediated transcription factors (Ascl1, Pou3f2, Myt1l, and Neurod1)	Neurons (Predominantly glutamatergic neurons)	Fibroblasts were converted to glutamatergic neurons *in vitro*	Pang et al., 2011
Pig	Lentivirus-mediated miR-124 and miR-9/9* combined with Ascl1	glutamatergic neurons and GABAergic neurons	Fibroblasts were converted to glutamatergic neurons and GABAergic neurons *in vitro*	Habekost et al., 2020
Mouse and human	Mouse: Lentivirus-mediated transcription factors (Ascl1, Pou3f2, Myt1l, Lhx3, Hb9, Isl1, and Ngn2) Human: Lentivirus-mediated transcription factors (Ascl1, Pou3f2, Myt1l, Lhx3, Hb9, Isl1, Ngn2, and Neurod1)	Motor neurons	Fibroblasts were converted to motor neurons (formed synapses and had action potentials) *in vitro*; The fibroblast-converted motor neurons integrated into the spinal cord after transplantation *in vivo*	Son et al., 2011
Mouse	Lentivirus-mediated transcription factors (Isl1 and Ngn2)	Motor neurons	Fibroblasts were converted to motor neurons (with spontaneous postsynaptic currents and repetitive action potentials) *in vitro*	Zhou et al., 2021
Human	Lentivirus-mediated transcription factor Oct4 and further induction with Lhx3	Motor neurons	Fibroblasts were converted to motor neurons (based on transcriptional profiles, electrophysiological function, synaptic activity, and the formation of neuromuscular junctions) *in vitro*; Motor function recovery was promoted after transplanting these fibroblast-converted motor neurons in rodent SCI models	Lee et al., 2020
Human	Lentivirus-mediated miR-124 and miR-9/9* combined with Isl1 and Lhx3	Motor neurons	Fibroblasts were converted to motor neurons *in vitro*	Abernathy et al., 2017
Human and mouse	Small molecules (kenpaullone, Forskolin, Y-27632, PMA, and RA)	Motor neurons	Fibroblasts were converted to motor neurons *in vitro* Motor neurons were detected in the dorsal skin of adult mice after transplantation of AG1-X2 beads (with five small molecules) *in vivo*	Qin et al., 2018
Human	Lentivirus-mediated transcription factors (Ngn2, Sox11, Isl1, and Lhx3), forskolin and dorsomorphin	Motor neurons	Fibroblasts were converted to motor neurons (on the basis of cytological and electrophysiological properties and the formation of functional neuromuscular junctions with skeletal muscle) *in vitro*	Liu et al., 2016
Mouse	Lentivirus-mediated transcription factor combination I (Olig1, Olig2, Nkx2.2, Nkx6.2, Sox10, ST18, Gm98, and Myt1) Lentivirus-mediated transcription factor combination II (Nkx6.2, Sox10, and Olig2)	Oligodendrocyte progenitor cells	Fibroblasts were converted to oligodendrocyte progenitor cells (on the basis of bipolar morphology and gene expression profiles) *in vitro*; Host axons were encapsulated, and dense myelin sheaths were produced *in vivo* in hypomyelinating mice after transplanting this fibroblast-converted oligodendrocyte progenitor cells	Najm et al., 2013
Mouse	Retrovirus-mediated transcription factor Oct4	Oligodendrocyte progenitor cells	Fibroblasts were converted to oligodendrocyte progenitor cells *in vitro*; functional recovery was promoted, and the risk of tumor formation was reduced after transplanting *in vivo* in an SCI model	Kim et al., 2015
Mouse	Retrovirus-mediated transcription factors (Pou3f4, Sox2, Klf4, c-Myc, and Tcf3) and the small molecule PDGF-AA	Oligodendrocyte progenitor cells	Fibroblasts were converted to oligodendrocyte progenitor cells *in vitro*; the recovery of motor function and expression of myelin basic protein was promoted after transplanting *in vivo* in an SCI model	Lee et al., 2018

### Neurons

#### Small molecule-based reprogramming of fibroblasts to neurons

The CFLSSVY combination of small molecules [CHIR99021, forskolin, LDN193189, SB431542, SP600125, valproic acid (VPA), and Y-27632] can induce the conversion of rat dermal fibroblasts into neurons *in vitro*. The induced neurons were grown on a 3D porous silk fiber scaffold to form a neural network and functional neural scaffold. Transplantation of this neural scaffold into rats with transection SCI reduced the size of the cavity, promoted axonal regeneration and myelination, repaired injured tissue, promoted motor nerve conduction, and improved hindlimb motor function (Hu et al., 2019).

Glutamatergic neurons and GABAergic neurons play important roles in the transmission of neurotransmitters in the spinal cord and influence spinal cord repair (Flynn et al., 2011; Bertels et al., 2022). The alternating addition of two combinations of small molecules, CLRDPAI (CHIR99021, LDN193189, RG108, dorsomorphin, P7C3-A20, A83-01, and ISX9) and FYDPAPP [forskolin, Y27632, DAPT, PD0325901, A83-01, purmorphamine (PMA), and P7C3-A20], to the induction medium could induce the conversion of human fibroblasts to glutamatergic neuron-like cells *in vitro*. The fibroblast-derived glutamatergic neuron-like cells had neuron-specific transcriptional profiles, displayed action potentials and could form functional synapses. Moreover, after transplantation into the brains of postnatal mice, fibroblast-derived glutamatergic neuron-like cells integrated into local neuronal circuits (Yang et al., 2019).

#### Transcription factor-based reprogramming of fibroblasts to neurons

*In vitro*, the combination of three neuron-specific transcription factors, achaete-scute family bHLH transcription factor 1 (Ascl1), POU class 3 homeobox 2 (Pou3f2) and myelin transcription factor 1 like (Myt1l), is sufficient to reprogram mouse embryonic fibroblasts and postnatal tail-tip fibroblasts into neurons, including some glutamatergic neurons and GABAergic neurons. These fibroblast-derived neurons generate action potentials and form functional synapses (Vierbuchen et al., 2010). In addition, human postnatal fibroblasts can be reprogrammed into glutamatergic neurons *in vitro* using four transcription factors, Ascl1, Pou3f2, Myt1l, and neuronal differentiation 1 (Neurod1) (Pang et al., 2011).

#### MicroRNA combination and transcription factor-based reprogramming of fibroblasts to neurons

Combinations of brain-enriched neurogenic microRNAs (miRNAs), such as miR-124 and miR-9/9*, significantly mediate the direct transformation of adult fibroblasts into functional neurons and can increase the efficiency of this transformation. During the direct conversion of mature fibroblasts into neurons, this combination can contribute to neurogenesis and inhibit fibroblast progression by altering the chromatin structure of cells (Abernathy et al., 2017) or maintaining fibroblasts in a neurogenic state (Lu and Yoo, 2018; Habekost et al., 2020). The combination of miR-124, miR-9/9* and Ascl1 can reprogram porcine fibroblasts into glutamatergic neurons and GABAergic neurons *in vitro*. During this reprogramming process, fibroblast-specific genes are silenced, whereas neuron-specific genes are activated (Habekost et al., 2020).

#### Transcription factor-based reprogramming of fibroblasts to motor neurons

Motor neurons are specialized neurons located in the anterior horn of the spinal cord that innervate the skeletal muscle and control its movement. Degeneration or loss of motor neurons is a typical feature of SCI (Qin et al., 2018; Rajpurohit et al., 2020).

Neuron-specific transcription factors, including Ascl1, Pou3f2, and Myt1l, and the motor neuron-specific transcription factors LIM homeobox 3 (Lhx3), motor neuron and pancreas homeobox 1 (Hb9), ISL LIM homeobox 1 (Isl1) and transcription factor neurogenin 2 (Ngn2) can directly transform mouse fibroblasts into motor neurons with high efficiency *in vitro*. Similarly, human-derived fibroblasts can be converted into motor neurons by these seven factors and Neurod1. When treated with these 7 factors, mouse fibroblast-derived motor neurons skip the neural progenitor phase. The resulting cells not only exhibit the gene expression profiles of motoneurons but can also form functional synapses with muscles and generate repetitive action potentials. *In vivo*, these reprogrammed motor neurons can efficiently integrate into the spinal cord when transplanted into the ventral cavity of the neural tube in chick embryos (Son et al., 2011).

Mouse embryonic fibroblasts treated with the transcription factors Ngn2 and Isl1 can be directly reprogrammed into motor neurons with spontaneous postsynaptic currents and repetitive action potentials *in vitro* (Zhou et al., 2021). In addition to these two factors, the sequential addition of organic cation/carnitine transporter 4 (Oct4) and Lhx3 can convert human-derived fibroblasts into functional motor neurons *in vitro*. Transplantation of Oct4- and Lhx3-treated motor neurons into rodents resulted in significant recovery of motor function following SCI (Lee et al., 2020). Both of these methods use only two transcription factors to induce somatic cell reprogramming, reducing the chance of viral mutation and simplifying the process (An et al., 2019).

#### MicroRNA combination and transcription factor-based reprogramming of fibroblasts to motor neurons

*In vitro*, miR-9/9* and miR-124 combine with the motor neuron transcription factors Isl1 and Lhx3 and can directly transform fibroblasts into functionally mature motor neurons (Abernathy et al., 2017). Notably, the motor neuron-specific transcription factors Isl1 and Lhx3 were used in the four abovementioned studies (Son et al., 2011; Abernathy et al., 2017; Lee et al., 2020; Zhou et al., 2021), suggesting a key role for these two transcription factors in the conversion of fibroblasts into motor neurons (Erb et al., 2017).

#### Small molecule-based reprogramming of fibroblasts to motor neurons

In addition to the above methods, small molecule-mediated cellular reprogramming has been shown to be effective. The small molecule combination KFYPR, comprising kenpaulone, forskolin, Y-27632, PMA, and retinoic acid (RA), was found to effectively downregulate the expression of fibroblast-specific genes and upregulate the expression of neuron-specific genes, thereby converting mouse and human fibroblasts into motor neurons and bypassing the neural progenitor stage *in vitro*. Moreover, the transplantation of highly concentrated KFYPR-treated AG1-X2 beads into the dorsal subcutis of adult mice can result in the production of motor neurons in percutaneous tissue, suggesting that the small molecule combination KFYPR can induce motor neurogenesis *in vivo* (Qin et al., 2018).

#### Combination of transcription factor and small molecule-based reprogramming of fibroblasts to motor neurons

Important progress has been made in the development of transcription factor- or small molecule-mediated fibroblast reprogramming strategies for repairing the injured spinal cord. However, it remains unclear whether combining transcription factors and small molecules could be more effective for fibroblast reprogramming. The combination of the transcription factors Ngn2, SRY-box transcription factor 11 (Sox11), Isl1, and Lhx3 and the small molecules forskolin and dorsomorphin has been found to directly transform adult skin fibroblasts into motor neurons. The transformed cells were shown to have the electrophysiological characteristics of mature spinal motor neurons, form neuromuscular junctions with skeletal muscle and control muscle activity. These researchers also used the transcription factors Isl1 and Lhx3, indicating that these factors may be key for reprogramming fibroblasts into motor neurons (Liu et al., 2016).

### Oligodendrocytes

SCI causes demyelination of myelinated nerve fibers, creating an obstacle to spinal cord repair, for which myelin regeneration is critical. Oligodendrocytes are mainly involved in the production and maintenance of myelin sheaths in the CNS (Chanoumidou et al., 2021). Myelinated oligodendrocytes in the CNS are generated from oligodendrocyte progenitor cells (Butt et al., 2019). Therefore, oligodendrocyte progenitor cells are ideal therapeutic cells for treating SCI and demyelination-related diseases.

#### Transcription factor-based reprogramming of fibroblasts to oligodendrocytes

Inducing the expression of factors such as oligodendrocyte transcription factor 1 (Olig1), oligodendrocyte transcription factor 2 (Olig2), NK2 homeobox 2 (Nkx2.2), NK6 homeobox 2 (Nkx6.2), SRY box transcription factor 10 (Sox10), ST18 C2H2C-type zinc finger transcription factor (ST18), myelin regulatory factor (Gm98), myelin transcription factor 1 (Myt1) or the combination of the transcription factors Nkx6.2, Sox10, and Olig2 can transform mouse embryos and lung fibroblasts into oligodendrocyte progenitor cells *in vitro*. These oligodendrocyte progenitor cells exhibit an oligodendrocyte-like bipolar morphology and gene expression profile and could be cultured to the fifth generation. Furthermore, following transplantation into hypomyelinated mice, these cells can survive and wrap around host axons to further produce dense myelin sheaths (Najm et al., 2013).

It has also been reported that ectopic expression of Oct4 can directly transform adult mouse fibroblasts into self-renewing, amplifiable bipotent oligodendrocyte progenitor cells *in vitro* under specific conditions. Oct4-induced oligodendrocyte progenitor cells propagate *in vitro* with more than 31-fold higher efficiency. Transplantation of these cells into mice effectively promotes functional recovery after SCI and reduces tumorigenic risk by reducing host genome modification (Kim et al., 2015).

#### Combination of transcription factor and small molecule-based reprogramming of fibroblasts to oligodendrocytes

The combination of the transcription factors POU class 3 homeobox 4 (Pou3f4), SRY-box transcription factor 2 (Sox2), Kruppel-like factor 4 (Klf4), Myc-like transcriptional regulator Myc-like (c-Myc), and transcription factor 3 (Tcf3) with the small molecule PDGF-AA has been shown to reprogram mouse fibroblasts into oligodendrocyte progenitor cells *via* a two-step process. This approach first induces the direct transformation of fibroblasts into neural stem cells; subsequently, these cells are differentiated into oligodendrocyte progenitor cells that proliferate and efficiently differentiate into oligodendrocytes. Moreover, transplantation of these induced oligodendrocyte progenitor cells into SCI rats can promote the expression of myelin basic protein in the spinal cord and reduce the inflammatory response after SCI to some extent, ultimately promoting the recovery of motor function (Lee et al., 2018).

In summary, fibroblast reprogramming strategies are important approaches for spinal cord repair. Although most studies on fibroblast reprogramming into motor neurons were conducted *in vitro*, the results have established a solid foundation for using fibroblast reprogramming strategies *in vivo*. With further advancements, this technique could be used to overcome more challenges in spinal cord repair. In the abovementioned *in vivo* transplanted studies, neurons, motor neurons, and oligodendrocyte progenitor cells obtained from fibroblasts contributed to spinal cord repair and promoted functional recovery, indicating that transplantation of fibroblast-derived cells is a potential and promising therapy for SCI (Kim et al., 2015; Hu et al., 2019; Lee et al., 2020). However, the mechanism of fibroblast reprogramming has not been thoroughly discussed in the above reports. Single-cell-based deep sequencing technology may promote the exploration of the mechanisms of fibroblast reprogramming in the future (Shin et al., 2015). Moreover, more research is needed to determine the optimal number of transplanted fibroblast-derived cells, improve the survival rate of transplanted cells and enhance their functional integration with host circuits.

## *In vivo* astrocyte reprogramming

After SCI, astrocytes, fibroblasts, microglia, NG2 cells, ventricular canal cells and pericytes are activated, and they proliferate, migrate and generate fibroglial scars in and around the damaged area. Irreversible neuronal loss occurs at the injury site (Yates et al., 2019; Dias et al., 2021). Glial scars, of which reactive astrocytes are a major component, are thought to play a dual role in spinal cord repair (Adams and Gallo, 2018; Yang et al., 2020a; Gilbert et al., 2022), and it is now believed that a modest reduction in glial scar density is important for repair of the injured spinal cord (Hackett et al., 2018; Hesp et al., 2018; Yang et al., 2020b).

Astrocytes can be activated to form neurospheres when isolated and cultured *in vitro*. Several studies have found that astrocytes can be successfully reprogrammed into neurons by specific transcription factors or small molecule combinations (Wang et al., 2020). For instance, forced expression of the transcription factor Ngn2 and distal-free homolog 2 (Dlx2) can reprogram reactive astrocytes into glutamatergic neurons and GABAergic neurons, respectively (Heinrich et al., 2010), illustrating the differentiation potential of astrocytes and suggesting that endogenous astrocytes are a good choice for *in vivo* reprogramming studies. Astrocytes are widely distributed throughout the spinal cord, undergo reactive proliferation after SCI and are highly plastic. Therefore, the transformation of astrocytes into target cells, including neurons, motor neurons, other subtypes of neurons and oligodendrocytes, seems to be a promising approach for spinal cord repair (Chen and Li, 2022; Table 2).

**TABLE 2:** Studies on the therapeutic effect of *in vivo* astrocyte reprogramming on SCI.

Subject	Induction method	Cell type	Achievements	References
Mouse	Lentivirus-mediated transcription factor (Sox2) with the small molecule VPA	Neurons and GABAergic-like neurons	*In situ* reprogramming of astrocytes into neurons was achieved *in vivo*; further maturation of neurons was promoted combined with VPA, and the formation of GABAergic-like neurons was induced *in vivo* in the spinal cord	Su et al., 2014
Mouse	Lentivirus-mediated transcription factor (Sox2) with shRNA-P53	Neurons (predominantly glutamatergic neurons)	The number of neurons in the ventral part of the mouse spinal cord (predominantly glutamatergic neurons) was increased; the number of neurons near the injury site was significantly increased in an SCI model	Wang et al., 2016
Mouse	AAV-mediated Transcription factor (Sox2)	Neurons	Astrocytes were converted to neurons *in vivo* and, combined with rehabilitation therapy, promoted motor function recovery in mice with crush spinal cord injury	Yang et al., 2020b
Rat	Lentivirus-mediated transcription factor (Zfp521)	Neurons	*In situ* reprogramming of astrocytes into neurons was achieved *in vivo*; motor function recovery was promoted and motor neuron-evoked potentials were observed in an SCI model	Zarei-Kheirabadi et al., 2019
Mouse	Transcription factors (Oct4 and Klf4) (transgenic mice)	Neural stem cell-like cells	Astrocytes were converted to neural stem cell-like cells *in vitro*; the porosity of scar tissue at the lesion site was increased, promoting myelin regeneration and motor function recovery in a contusion SCI mouse model	Huang et al., 2020
Mouse	AAV-mediated transcription factor (Ngn2)	Glutamatergic neurons and GABAergic neurons	*In vivo*, astrocytes were converted to glutamatergic neurons and GABAergic neurons; responding to different afferent inputs from the dorsal root ganglion; astrocytes were converted to neurons in an SCI model	Liu et al., 2021
Mouse	AAV-mediated transcription factor (Neurod1)	Neurons (predominantly glutamatergic neurons predominate); when combined with Dlx2, Neurod1 increased the percentage of GABAergic neurons	In a stab wound SCI model, *in situ* reprogramming of astrocytes to glutamatergic neurons was achieved; integration into local spinal circuits by generating repetitive action potentials and spontaneous synaptic responses	Puls et al., 2020
Mouse	Lentivirus-mediated transcription factors (Ngn2 and Isl1) *in vitro*; AAV-mediated Ngn2 and Isl1 *in vivo*	Motor neurons	Astrocytes were converted to motor neurons (with spontaneous postsynaptic currents and repetitive action potentials) *in vitro; in vivo*, spinal astrocytes can be reprogrammed into motor neurons in the adult mouse	Zhou et al., 2021
Mouse	Lentivirus-mediated shRNA-PTB *in vitro*; AAV-mediated shRNA-PTB *in vivo*	Motoneuron-like cells	Astrocytes were converted to motoneuron-like cells *in vitro*; *in vivo*, the density of glia scar tissue at the lesion site was decreased, motoneuron-like cells were replenished around the lesion site, and motor function recovery was promoted in a compression SCI mouse model	Yang et al., 2023
Rat	Protein (Nrg1)	Oligodendrocyte lineage cells	Astrocytes were converted to oligodendrocyte lineage cells *in vitro*; in an SCI model, astrocytes were converted to oligodendrocyte lineage cells; astrocyte proliferation was inhibited, myelin regeneration was promoted, axons were protected, and recovery of motor function was promoted	Ding et al., 2021

### Neurons

#### Combinations of transcription factors and small molecules for reprogramming astrocytes into neurons

Sox2 is an important transcription factor for astrocyte reprogramming. Doublecortin (Dcx), a microtubule-associated protein, is abundantly expressed in neuronal cells (Zarei-Kheirabadi et al., 2019). The overexpression of Sox2 was sufficient to reprogram endogenous spinal cord astrocytes into Dcx-positive neuroblasts. Such neuroblasts can be further transformed into synaptogenic neurons *in situ* (Su et al., 2014). When combined with other small molecules, Sox2 further induces the reprogramming of astrocytes into specific neuronal subtypes. Histone deacetylases play an important role in chromatin remodeling and epigenetic regulation, and their inhibitors can access chromatin to promote the transcription of silenced genes (Zhao et al., 2020). VPA, a histone deacetylase inhibitor, can increase cell reprogramming efficiency and promote normal neurogenesis and the maturation of neuroblasts (Yoshida et al., 2021). Studies have shown that applying the transcription factor Sox2 combined with VPA can further convert neurons into GABAergic-like neurons *in vivo* (Su et al., 2014).

#### Combination of transcription factor and gene knockdown-based reprogramming of astrocytes to neurons

Sox2 combined with shRNA-P53 was found to induce the conversion of astrocytes into glutamatergic neuron-dominated neurons *in situ*, significantly increasing the number of neurons in the ventral part of the mouse spinal cord. The researchers further applied this combination in mice with contusion SCI and found that it could significantly increase the number of neurons around the lesion site (Wang et al., 2016). This is because Sox2-mediated intrinsic neurogenesis in the adult mouse spinal cord occurs *via* glial cell reprogramming and is dependent on the p53 pathway, while silencing of p53 significantly promotes Sox2-mediated astrocytic reprogramming.

#### Transcription factor-based reprogramming of astrocytes to neurons

Induction of Sox2 expression combined with rehabilitation can significantly improve functional recovery in SCI mice (Yang et al., 2020b). These findings suggest that astrocyte reprogramming combined with rehabilitation could be an effective strategy to promote spinal cord repair.

In addition to Sox2, many other factors are involved in astrocyte reprogramming. It has been reported that the transcription factor zinc finger protein 521 (Zfp521) can reprogram astrocytes into neurons through the progenitor cell stage *in situ* in the spinal cords of rats with contusion SCI. These reprogrammed astrocytes were found to significantly improve motor performance and exhibit motor evoked potentials *in vivo* (Zarei-Kheirabadi et al., 2019). *In vitro*, the transcription factors Oct4 and Kruppel-like factor 4 (Klf4) were shown to transform primitive astrocytes into neural stem-like cells. In a contusion SCI mouse model, the combination of these two transcription factors effectively increased the porosity of glial scar tissue at the injury site, promoting myelin regeneration in axons after injury and improving the recovery of motor function (Huang et al., 2020).

Ngn2 is an essential transcription factor for the expression of glutamatergic neurotransmitter phenotypes in the embryonic neocortex and is critical for cell reprogramming (Falco et al., 2021). Ngn2 reprogrammed astrocytes into glutamatergic neurons and GABAergic neurons *in situ* in the dorsal spinal cords of adult mice. Reprogrammed neurons can integrate into local neural circuits and can respond to different afferent inputs from the dorsal root ganglia. Ngn2 has also been found to reprogram astrocytes into neurons at the site of injury in SCI models (Liu et al., 2021). Neurod1, a downstream target of Ngn2, can reprogram astrocytes in the dorsal horn of the spinal cord into glutamatergic neurons at the injury site *in situ* in a mouse model of SCI resulting from stab injury. These reprogrammed neurons can generate repetitive action potentials and spontaneous synaptic responses and thereby integrate into local spinal circuits. In addition, Neurod1 combined with the transcription factor Dlx2 significantly increases the proportion of astrocytes that are reprogrammed into GABAergic neurons at the site of SCI (Puls et al., 2020).

#### Transcription factor-based reprogramming of astrocytes to motor neurons

Because of the abundance of astrocytes in the spinal cord, the direct conversion of astrocytes to motor neurons *in situ* is important for nerve regeneration and functional recovery after SCI. CRISPR-mediated activation of the endogenous genes Ngn2 and Isl1 was shown to enable *in vitro* the reprogramming of mouse spinal cord astrocytes into motor neurons with spontaneous synapses and repetitive action potentials. *In vivo* experiments also revealed that an increase in Ngn2 and Isl1 expression can reprogram spinal astrocytes into motor neurons and that the axons of these motor neurons can project to the sciatic nerve (Zhou et al., 2021).

#### Gene knockdown-based reprogramming of astrocytes to motor neurons

Extensive motor neuron loss occurs at the lesion site after SCI and astrocytes proliferate and form dense glial scars. Regulating the density of glial scars and replenishing motor neurons in the injured area is essential for spinal cord repair. Polypyrimidine tract binding protein (PTB) is a ribonucleic acid-binding protein involved in neurogenesis. To date, several laboratories have demonstrated that PTB silencing can effectively reprogram brain-derived astrocytes into functional neurons *in vitro* (Qian et al., 2020; Maimon et al., 2021). Moreover, in mouse models of Parkinson’s disease and aging, the knockdown of PTB can replenish dopaminergic and new neurons in the brain, respectively, ultimately promoting functional recovery in mice. Furthermore, silencing PTB can replenish ganglion cells in the mouse retina and promote the recovery of visual responses in mice with visual impairment (Zhou et al., 2020).

However, an interesting challenge was recently noted in this field (Wang L. L. et al., 2021). It has been argued that silencing PTB (Qian et al., 2020) cannot induce the rapid reprogramming of brain astrocytes into neurons *in situ*. It was concluded that the replenished neurons observed *in vivo* originated from endogenous neurons and were not reprogrammed brain astrocytes. Similarly, some researchers believe that astrocyte reprogramming is a long and gradual developmental process *in vivo* and that the conversion rate might be related to virus concentration and type. The origin of the replenished neurons cannot be determined through currently available biotechnological tools. However, it is clear that these neurons can promote functional recovery in disease models. Therefore, we recently explored the role of PTB silencing in spinal cord repair.

We discovered for the first time that mouse spinal cord reactive astrocytes can be successfully reprogrammed into motoneuron-like cells by knocking down the expression of PTB using lentivirus-expressing shRNA and antisense oligonucleotides *in vitro*. In a mouse model of compression SCI, PTB knockdown moderately reduced the density of the glial scar without destroying its overall structure while increasing the number of motoneuron-like cells around the injured area, effectively reducing cell apoptosis around the injured area and ultimately promoting the recovery of motor function in SCI mice (Yang et al., 2023). Based on the current literature, we hypothesize that the replenished motoneuron-like cells observed in the spinal cords of SCI mice after PTB silencing may either be induced by *in situ* astrocytic reprogramming, originate from endogenous neurons due to the protective effect of PTB silencing on neurons, or are derived from PTB silencing-mediated resident neural stem cells differentiation. Although the source of the replenished neurons needs to be confirmed in further studies, it is clear that PTB silencing promotes motor function recovery in SCI mice. These results suggest that PTB silencing may be a promising therapeutic approach for SCI.

### Oligodendrocytes

#### Protein-based reprogramming of astrocytes to oligodendrocytes

SCI can lead to neuronal necrosis and axonal demyelination (Zhou P. et al., 2019), hindering myelin regeneration and the replacement of lost neurons and motor neurons. Oligodendrocytes, the main cells forming myelin sheaths around axons (Xia and Fancy, 2021), also undergo degeneration after SCI, further leading to axonal demyelination (Pukos et al., 2019). Therefore, replenishing new oligodendrocytes is an effective strategy to promote remyelination after SCI (Plemel et al., 2014; Huntemer-Silveira et al., 2020). Conversion of astrocytes to oligodendrocytes could be an innovative therapeutic approach for SCI repair. The protein neuregulin-1 (Nrg1) was reported to directly reprogram tumor necrosis factor-alpha (TNF-α)-expressing reactive astrocytes into oligodendrocyte lineage cells *in vitro*. In animal models, intrathecal injection of Nrg1 was also found to convert reactive astrocytes into oligodendrocyte lineage cells, promote axonal remyelination and effectively inhibit astrocyte proliferation. This method was demonstrated to protect axons and further promote the recovery of motor function following injury (Ding et al., 2021). The study also suggested that converting astrocytes into oligodendrocytes facilitated myelin regeneration and effectively promoted spinal cord repair.

These above astrocyte reprogramming strategies convert astrocytes into mainly glutamatergic, GABAergic, or mature neurons. Only two groups have reported that astrocytes can be reprogrammed into motor neurons (Zhou et al., 2021; Yang et al., 2023). The identification of new key transcription factors or genes involved in astrocyte-derived motor neuron reprogramming is expected to be important in motor function recovery after SCI.

The region-specific status of astrocytes influences the fate of neuronal subtypes in cell reprogramming. Astrocytes in specific regions have specific transcriptional and proteomic environments that lead to reprogramming into different subtype-specific neurons (Mattugini et al., 2019; Herrero-Navarro et al., 2021; Kempf et al., 2021), but the specific mechanisms are unclear and require further exploration. The functional recovery of SCI by astrocyte reprogramming depends on how many functional neurons are replenished *in situ*, whether they are the appropriate neuronal subtypes, and whether they can integrate into the right neural circuits.

## *In vivo* NG2 cell reprogramming

Recent research has shown that NG2 cells act as a progenitor cell pool and can be reprogrammed into oligodendrocytes, astrocytes and even neurons under specific treatment conditions (Valny et al., 2017; Sanchez-Gonzalez et al., 2020; Chen et al., 2021). In brain injury and neurodegenerative disease models, retroviral infection of NG2 cells with the single transcription factor Neurod1 reprograms these cells *in situ* into functional neurons, such as glutamatergic and GABAergic neurons, promoting functional recovery (Guo et al., 2014). These results illustrate the strong multidifferentiation potential and plasticity of NG2 cells in spinal cord repair (Table 3).

**TABLE 3:** Studies on the therapeutic effect of *in vivo* NG2 cell and neural progenitor cell reprogramming on SCI.

Somatic cell	Subject	Induction method	Cell type	Achievements	References
NG2 cell	Mouse	PD168393 (EGF receptor inhibitor)	Neurons	NG2 cells were converted to neurons *in vitro*; the number of neurons was increased, and the neurobehavioral performance of SCI model mice was improved	Ju et al., 2012
NG2 cell	Mouse	Lentivirus-mediated transcription factor (Sox2)	Neurons	In an SCI model, NG2 cells were converted to mature neurons, formed synaptic connections, reduced scar tissue formation and promoted functional recovery	Tai et al., 2021
Neural progenitor cell	Rat	Retrovirus-mediated transcription factors (Ascl1 and Ngn2) combined with growth factors	Neurons (Ngn2 with growth factor); oligodendrocytes (Ascl1 with growth factor)	In an SCI model, neural progenitor cells were converted to neurons (mainly GABAergic neurons) and oligodendrocytes *in situ*	Ohori et al., 2006

### Chemical reprogramming of NG2 cells into neurons

The epidermal growth factor (EGF) receptor inhibitor PD168393 has been reported to cause NG2 cells to acquire a neuronal phenotype. PD16839 was shown to reprogram NG2 cells directly into neurons in a contusion SCI mouse model, significantly increasing the number of neurons at the injury site and effectively improving neurobehavioral performance after injury (Ju et al., 2012).

### Transcription factor-based reprogramming of NG2 cells to neurons

High ectopic expression of Sox2 can lead to the reprogramming of NG2 cells into mature spinal cord neurons that can form synaptic connections with endogenous neurons in the spinal cord in SCI mouse models. These induced spinal cord neurons undergo neuronal conversion around the lesion site and form connections to achieve spontaneous functional recovery. Moreover, they effectively reduce the formation of glial scars after SCI and promote functional recovery in mice (Tai et al., 2021).

The above studies show the substantial potential of NG2 cell reprogramming in spinal cord repair. It has been demonstrated previously that inhibition of NG2 cells can promote the repair of SCI (Rodriguez et al., 2014; Hesp et al., 2018). Therefore, we wondered whether reprogramming with NG2 cells as target cells leads to a decrease in NG2 cells, further promoting SCI repair. These questions deserve further study.

## *In vivo* neural progenitor cell transformation

Numerous studies have revealed that neural progenitor cells transplanted into various animal models can be induced to differentiate into target cells using specific approaches and that these cells promote functional recovery (Xue et al., 2021). Although the differentiation of endogenous neural progenitor cells into neurons and oligodendrocytes is strictly regulated by the cellular environment (Asrican et al., 2020), these cells can be applied for spinal cord repair (Table 3).

### Combination of transcription factor and growth factor-based reprogramming of neural progenitor cells to neurons or oligodendrocytes

Some studies in rat SCI models have demonstrated that viral-mediated delivery of the transcription factor Ngn2 into the injury site, combined with EGF and fibroblast growth factor 2 (FGF2) can transform spinal endogenous neural progenitor cells into mainly GABAergic neurons *in situ*. The transcription factor Ascl1 combined with growth factors was shown to convert spinal cord endogenous neural progenitor cells into oligodendrocytes *in situ* (Ohori et al., 2006). However, in this study, the number of neural progenitor cell-derived neurons or oligodendrocytes was insufficient for functional repair in SCI.

Currently, SCI treatment is limited by the restricted differentiation of endogenous neural progenitors *in vivo*. Thus, the transformation of endogenous neural progenitor cells into neurons could be a novel and effective treatment strategy for spinal cord repair if these cells meet the requirements of specific neuronal subtypes and oligodendrocytes after injury (Voronova et al., 2020a).

## Comparison of different somatic cell reprogramming methods to repair spinal cord injury

In previous studies, fibroblast-derived neurons or oligodendrocyte progenitor cells were transplanted into the spinal cord for spinal cord repair (Kim et al., 2015; Lee et al., 2020). The use of external cells for fibroblast reprogramming therapy for spinal cord repair poses some difficult challenges. Reprogrammed cell transplantation is also complicated by immune rejection and poor cell purity and survival (Chen et al., 2015). Moreover, the process of cell transplantation itself causes damage to nerve tissue. Compared with fibroblast reprogramming, astrocyte reprogramming for spinal cord repair has the important advantage of converting spinal cord astrocytes into neurons *in situ.* Astrocyte reprogramming makes use of endogenous activated astrocytes that are adjacent to the lost neurons to regenerate functional cells in the injured area after SCI (Zarei-Kheirabadi et al., 2019; Puls et al., 2020). In addition to astrocytes, NG2 cells and neural progenitors can be reprogrammed into neurons *in situ.* However, there are fewer residual NG2 cells and neural progenitors than astrocytes around the injured site after SCI; thus, NG2 cell and neural progenitor reprogramming-mediated spinal cord repair is relatively difficult (Chen and Li, 2022). Reactive astrocytes are an abundant cell source for endogenous somatic reprogramming due to their ability to proliferate after SCI (Li and Chen, 2016). The graft-free somatic cell reprogramming strategy holds substantial promise for clinical SCI repair therapies.

## Challenges and controversies

Although recent progress has been made in the application of somatic cell reprogramming technology for spinal cord repair in preclinical studies, there are still some challenges in translating this strategy to clinical settings for SCI treatment. First, safety remains the most important issue related to somatic cell reprogramming for SCI treatment. Some transcription factors that induce somatic reprogramming are also highly expressed in some tumors. For instance, many studies have revealed that Sox2 is overexpressed in squamous cell carcinomas in many tissues, including the paranasal sinuses, hypopharynx, larynx, and lung (Zhou Y. X. et al., 2019). Second, safer and more effective methods for delivering reprogramming factors should be validated. Transcription factor delivery is currently achieved *via* viral infection, in which viral vectors integrate into the host cell genome (Vignoles et al., 2019). Despite the efficacy of viral vectors, this approach is associated with risks, including insertional mutagenesis, transgene integration, cellular senescence, strong immunogenicity, and viral infection (Gurumoorthy et al., 2022). Third, in the small molecule-mediated conversion approach *in vivo*, cell type specificity is difficult to achieve, and toxicity is a challenge (Hu et al., 2018). Fourth, our mechanistic knowledge of the somatic cell reprogramming process is rather rudimentary. More research is required to explore the molecular and cellular mechanisms of somatic cell reprogramming (Wang and Zhang, 2018).

Although skin fibroblasts from patients can be used for fibroblast reprogramming strategies, which is a promising strategy for autologous transplantation, research is mostly limited to cellular studies and animal experiments. Therefore, the clinical application of fibroblast-derived cells for therapy may involve many challenges. How to determine the optimal number of transplanted cells, balance efficacy and safety, and improve the survival rate of transplanted cells and their functional integration with host circuits requires further exploration. Extensive studies are required in the future to improve the efficacy of fibroblast-derived cell transplantation for the clinical treatment of SCI. *In vivo* reprogramming is a very promising approach for treating SCI in clinical settings because it utilizes endogenous cells. The development of AAV-mediated gene therapy involving cell-type-restricted promoters makes it possible to target resident cells in the spinal cord, which is the advantage of *in situ* cell reprogramming. AAV-mediated therapies have been successfully translated to the clinic in some cases; for example, an AAV9 vector has been used to induce the expression of SMN1 in motor neurons for treating spinal muscular atrophy (Vasan et al., 2021). NG2 cells and neural progenitor cells can differentiate into glial scar-forming astrocytes after SCI (Zhu et al., 2008); thus, there are fewer residual NG2 cells and neural progenitor cells than astrocytes. Astrocytes are activated and proliferate after SCI; thus, these cells are an ideal cell source for *in vivo* reprogramming in clinical settings (Wang and Zhang, 2018).

Recently, a different view related to astrocyte reprogramming has emerged (Wang L. L. et al., 2021). In addition to the controversy associated with PTB silencing discussed above (Qian et al., 2020), it has been argued that overexpression of the transcription factor Neurod1 (Guo et al., 2014) cannot reprogram brain astrocytes into neurons *in situ*. The opposing view is that *in vivo* replenished neurons are derived from endogenous neurons rather than reprogrammed brain astrocytes. Although the origin of replenished neurons *in vivo* following PTB silencing or Neurod1 overexpression has not yet been conclusively determined, these neurons promote functional recovery in disease-model mice. Therefore, from the perspective of promoting functional recovery, somatic cell reprogramming technology still has substantial potential for promoting spinal cord repair.

The efficacy of somatic cell reprogramming treatment strategies in human clinical trials could be limited due to the lack of control groups, and improvements in function observed in the trials could be related to decompression surgery at the time of cell transplantation or natural recovery after SCI rather than therapeutic effects (Bartlett et al., 2020). Future studies are needed to confirm the efficacy of somatic cell reprogramming in human clinical trials to promote its clinical application for SCI repair (Voronova et al., 2020b). Moreover, the severity of SCI is determined by multiple factors that must be considered when determining the appropriate treatment. Using biomaterials as carriers of reprogrammed cells, viral vectors and/or small molecules can improve somatic cell reprogramming strategies to achieve optimal spinal cord repair (Pinelli et al., 2022). Future work is required to devise clinical treatments for somatic cell reprogramming combined with biomaterials *in vivo*.

## Conclusion

The development of somatic cell reprogramming approaches for spinal cord repair has progressed rapidly in recent years. However, due to the complexity of this process, the specific mechanisms require further investigation (Havelikova et al., 2022). Bypassing the iPSC phase during somatic cell reprogramming could avoid the potential risk of tumor formation. Furthermore, the direct transformation of somatic cells into neurons, functional neuronal subtypes or even oligodendrocytes is highly efficient, can be achieved quickly and is not limited by ethical issues, indicating that this approach shows promise as a potential new treatment for SCI. Somatic cell reprogramming technology is still in the early stages of development, and considerable investigation remains to be conducted before these approaches can be translated to clinical settings. Despite the limited data on functional recovery after SCI, available findings indicate that somatic cell reprogramming techniques have good prospects for spinal cord repair, cell regeneration, tissue repair, and functional recovery. We believe that developments in biotechnology and regenerative medicine and breakthroughs in somatic cell reprogramming technology will eventually lead to improvements in the efficacy of spinal cord repair methods.

## Author contributions

GC designed the review. RY wrote and drafted the manuscript. All authors contributed to the revision of the manuscript and approved the final version.
